# Implementing a behavioral medicine approach in physiotherapy for patients with musculoskeletal pain: a scoping review

**DOI:** 10.1097/PR9.0000000000000844

**Published:** 2020-09-23

**Authors:** Anne Söderlund, Maria Elvén, Maria Sandborgh, Johanna Fritz

**Affiliations:** School of Health, Care and Social Welfare, Mälardalen University, Västerås, Sweden

**Keywords:** Behavioral medicine, Physiotherapy, Implementation, Scoping review

## Abstract

In intervention research on musculoskeletal pain, physiotherapists often study behavioral and cognitive components. Evidence on applying these components has increased during the past decade. However, how to effectively integrate behavioral and cognitive components in the biopsychosocial management of musculoskeletal pain is challenging. The aim was to study the intervention components and patient outcomes of studies integrating behavioral and cognitive components in physiotherapy, to match the interventions with a definition of behavioral medicine in physiotherapy and to categorize the behavior change techniques targeted at patients with musculoskeletal pain in (1) randomized controlled effect trials or (2) implementation in clinical practice trials. A scoping review was used to conduct this study, and the PRISMA-ScR checklist was applied. Relevant studies were identified from the PubMed, MEDLINE, PsycINFO, CINAHL Plus, and Web of Science Core databases separately for the (1) randomized controlled effect trials and (2) implementation in clinical practice trials. Synthesis for the matching of the patient interventions with the existing definition of behavior medicine in physiotherapy showed that the interventions mostly integrated psychosocial, behavioral, and biomedical/physical aspects, and were thus quite consistent with the definition of behavioral medicine in physiotherapy. The reported behavior change techniques were few and were commonly in categories such as “information of natural consequences,” “feedback and monitoring,” and “goals and planning.” The patient outcomes for long-term follow-ups often showed positive effects. The results of this scoping review may inform future research, policies, and practice.

## Abstract 2

Supplemental Digital Content is Available in the Text.

The interventions in the scoping review were in agreement with the definition of behavioral medicine in physiotherapy, but reported behavior change techniques were few.

## 1. Introduction

Patients seeking for musculoskeletal pain care are common in physiotherapists' clinical practice. Modern pain management should be guided by a biopsychosocial theoretical approach,^14^ which is complex. In intervention research on musculoskeletal pain, physiotherapists often study variations of what are called “behavioral and cognitive components,” and positive sustainable evidence of applying these components has greatly increased during the past decade.^7,26,32,39,59–61^ However, how to effectively *integrate* the “behavioral and cognitive components” in the management of musculoskeletal pain from a biopsychosocial theoretical approach is challenging.

Behavioral medicine in physiotherapy, informed by the International Society for Behavioral Medicine's definition of behavioral medicine,^30^ ie, the “integration of psychosocial, behavioral and biomedical knowledge in analyses of patients'/clients' behaviors in activities of importance for participation and in choosing and applying treatment and behavior change methods and evaluating outcomes,”^47^ can guide us to a better integration of important behavioral, psychosocial, and physical components in all phases of patient encounters. According to the behavioral medicine approach, patients with musculoskeletal pain should be coached to self-manage, to change behavior when needed, and to reduce dependency on health care.^15,16,46,50^ A uniform description of how to *integrate* behavioral, psychosocial, and physical/biomedical knowledge into interventions in musculoskeletal research could better guide practitioners and researchers.

Moving from randomized controlled trials to implementing behavioral medicine in physiotherapy practice is a challenging task. A systematic review of physiotherapists' usage of behavior change techniques in promoting physical activity showed that only a small number of techniques were identified as being used in clinical practice.^32^ Furthermore, Fritz et al.^24^ studied the effects of multifaceted implementation methods on changing physiotherapists' clinical behavior when treating patients with musculoskeletal pain. They concluded that the methods could support the change in the short term, but that sustaining of the change needed different strategies and/or doses than those used in the study. Thus, although knowledge of the evidence-based behavior change techniques exists, the techniques are not well implemented, and psychosocial, behavioral, and biomedical knowledge is not optimally integrated in physiotherapy^12,13^ or specifically in management of musculoskeletal pain.^2,18^ A global common description for these interventions in musculoskeletal research could help practitioners and researchers to better communicate the treatments to patients, health care, and policymakers and thus set the stage for more effective implementation.

There are few studies that explicitly describe the integrative concept of behavior medicine in physiotherapy,^25,29,48^ and even fewer that describe its implementation.^18,24^ A scoping review would highlight the key components, identify the limitations of the current interventions using a behavioral medicine approach in physiotherapy, and provide direction for further research in the field.

The aim of the present scoping review was to study the intervention components and patient outcomes of studies integrating “behavioral and cognitive components” in physiotherapy, to match the interventions with a definition of behavioral medicine in physiotherapy, and to categorize the behavior change techniques targeted at patients with musculoskeletal pain in (1) randomized controlled effect trials or (2) implementation in clinical practice trials.

## 2. Methods

The systematic recommendations for a scoping review were used to conduct this study.^11,33^ In addition, the PRISMA-ScR checklist was used when reporting the results of this scoping review.^54,55^

This scoping review could not be registered in the PROSPERO registry due to current regulations of the registry. No formal protocol was written before conducting this review. However, an outline of the study was written and discussed between the authors before starting the study.

The methods are presented separately, when relevant, for the 2-fold aim of this study, ie, for the (1) randomized controlled effect trials and (2) implementation in clinical practice trials.

### 2.1. Eligibility criteria

To be included in this scoping review, the articles needed to meet the following inclusion criteria, separately for the (1) randomized controlled effect trials and (2) implementation in clinical practice trials.

Randomized controlled effect trials: peer-reviewed journal articles; patients with musculoskeletal pain; studies integrating physical, behavioral, and cognitive components in physiotherapy; and English language.

Implementation in clinical practice trials: quasiexperimental or experimental studies; patients with musculoskeletal pain; implementation of physical, behavioral, and cognitive components in physiotherapy in clinical practice; description of the implementation intervention on physiotherapists was provided (ie, what was the *implementation intervention* supporting the uptake of physiotherapists' new working approach); patient outcomes reported; English language, and peer-reviewed journal articles or unpublished manuscripts (through contact with identified authors).

### 2.2. Information sources and search strategy

To identify relevant studies, the combination of PubMed, MEDLINE, PsycINFO, CINAHL Plus, and Web of Science Core databases was searched on several occasions with the final search being performed on the 19th of October 2019 separately for (1) randomized controlled effect trials and (2) implementation in clinical practice trials. The searches were conducted with topic-relevant MeSH search terms. The full electronic search strategy, which was the same for all databases, is shown in Tables 1 and 2.

**Table 1:** Search strategy for behavioral medicine approach in physiotherapy regarding its effects on patient outcomes studied in randomized controlled trials for patients with musculoskeletal pain.

Databases (search date October 19, 2019) PubMed, MEDLINE, PsycINFO, CINAHL Plus, Web of Science Core
Search terms (“Behavioural medicine”[All fields] OR “behavioral medicine”[MeSH terms] OR (“behavioral”[All fields] AND “medicine”[All fields]) OR “behavioral medicine”[All fields]) AND (“physical therapy modalities”[MeSH terms] OR (“physical”[All fields] AND “therapy”[All fields] AND “modalities”[All fields]) OR “physical therapy modalities”[All fields] OR “physiotherapy”[All fields]) AND (“pain”[MeSH terms] OR “pain”[All fields]) AND (“random allocation”[MeSH terms] OR (“random”[All fields] AND “allocation”[All fields]) OR “random allocation”[All fields] OR “randomized”[All fields]) AND (“humans”[MeSH terms] AND English[lang])
No date-limits N = PubMed 148 hits + (CINAHL (6), MEDLINE (44), PsycINFO (16) + Web of Science Core (6)): Totally 220

**Table 2:** Search strategy for the impact on outcomes for patients with musculoskeletal pain of an implementation of behavioral medicine approach in physiotherapists' clinical practice.

Databases (search date October 19, 2019) PubMed, MEDLINE, PsycINFO, CINAHL Plus, Web of Science Core
Search terms (“Behavioural medicine”[All fields] OR “behavioral medicine”[MeSH terms] OR (“behavioral”[All fields] AND “medicine”[All fields]) OR “behavioral medicine”[All fields]) AND (“physical therapy modalities”[MeSH terms] OR (“physical”[All fields] AND “therapy”[All fields] AND “modalities”[All fields]) OR “physical therapy modalities”[All fields] OR “physiotherapy”[All fields]) AND (“pain”[MeSH terms] OR “pain”[All fields]) AND implementation[All fields] AND (“humans”[MeSH terms] AND English[lang])
No date-limits N = PubMed 19 hits + (CINAHL (1), MEDLINE (11), PsycINFO (4) + Web of Science Core (1)): Totally 36

### 2.3. Selection of sources of evidence

The first author conducted the database searches. The other authors contributed by identifying studies through other sources, ie, through personal contacts. All eligible studies' titles and abstracts were screened by the first author, who also decided which studies were included in the next step. If the decision for inclusion was perceived as uncertain, the study was included in the next step. In the next step, the full-text articles were downloaded to be assessed by all authors, and the decision for final inclusion was made in agreement.

### 2.4. Data charting process and parameters

Data were tabulated after jointly developing headings for the tables according to the aim of this scoping review. The data charting was divided between the authors, and finally, the correctness of the data in the finished tables was checked by all authors.

Table 3 for the randomized controlled effect trials includes the following: reference, country, aim, sample, experimental intervention, control intervention, and patient outcomes.

**Table 3:** Characteristics and patient outcomes of the included randomized controlled effect trials regarding investigations of a behavioral medicine approach in physiotherapy for patients with musculoskeletal pain.

Reference, Country	Aim	Sample	Experimental intervention	Control intervention	Results of patient outcomes
Archer et al.,^3^ USA	Study the effect of a cognitive–behavioral-based physical therapy program (CBPT) compared to an education program (EP).	Patients 6 wk after lumbar laminectomy, >21 y, n = 86	Standard care including advice about lifting and driving restrictions. CBPT program (in-person session and over the telephone) aimed to decrease fear of movement and increase self-efficacy, including behavioral self-management, problem solving, cognitive restructuring, and relaxation. Treatment manual was given.	Standard care including advice about lifting and driving restrictions. EP included sessions of benefits of physiotherapy, biomechanics after surgery, daily exercise, promoting healing, stress reduction, sleep, energy, communication with health care, and preventing injury.	For patients who had CBPT, disability and pain intensity decreased and physical function and general health increased significantly more compared to those with EP at 3 mo follow-up.
Bring et al.,^6^ Sweden	Study the effect of an individually tailored behavioral medicine approach in physiotherapy delivered through internet compared to same intervention delivered face-to-face or a control group having self-care instructions.	Patients with acute whiplash associated disorders, aged 18–65 y, n = 55	Individually tailored behavioral medicine intervention, based on functional behavioral analysis and everyday activity goals specifying physical, cognitive, and behavioral skills relevant for goal attainment. Enhancement of self-management skills and level of functioning, strategies for maintenance, and relapse prevention. Seven treatment modules were included.	Written self-care instructions about physical symptoms, relaxation, neck and shoulder range of motion exercises and daily walks.	Significant differences (favoring the individually tailored behavioral medicine intervention groups) between the groups over time (up to 12-mo follow-up) in disability, self-efficacy in activities, catastrophizing, and fear of movement, but not in pain intensity.
Cederbom et al.,^8^ Sweden	Study the effect of an individually tailored behavioral medicine approach in physiotherapy compared to one-time delivered advice on physical activity.	Older women with chronic musculoskeletal pain, aged > 65 y, n = 23	Behavioral medicine intervention integrated with physiotherapy. Individual functional behavior analysis of physical, psychological, social, and physical environmental factors affecting ability in specific everyday activities. Advice on physical activity and its benefits, goal setting, self-monitoring, feedback, problem-solving strategies, strategies for maintenance, and relapse prevention.	Standard care including one-time only advice on physical activity and its benefits.	No significant differences between groups in pain intensity, disability, or morale were found at any of the follow-ups (postintervention, 3 mo after intervention).
Cederbom et al.,^9,10^ Norway	Study the effects of an individually tailored behavioral medicine approach in physiotherapy compared to standard care.	Older persons, >75 y, with musculoskeletal pain, n = 105	Functional behavioral analyses of the physical, psychological, social, and environmental factors related to the goal behaviors and treatment goals. Improve physical, behavioral, cognitive, or social skills, improve self-efficacy, decrease fear of falling and fear of movement in the goal behavior, generalize the skills to other behaviors, strategies to maintain new behavior, and problem-solving strategies Advice on and increase of physical activity and its benefits, functional exercises, and self-monitoring of physical activity.	Standard care including one-time only advice on physical activity and its benefits.	There were differences in pain-related disability, pain severity, health-related quality of life, management of everyday activities, and self-efficacy in goal behaviors favoring the individually tailored behavioral medicine approach in physiotherapy intervention group. The effect on pain severity was maintained at 3-mo follow-up.
Hill et al.^27^ and treatment description by Main et al.,^36^ United Kingdom	Study the effects of a stratified (with the STarT back screening Tool classification) approach in management of low back pain in comparison to current best practice in primary care.	Patients with low back pain of any duration, mean age > 18 y, n = 851	All patients in intervention group: Assessment, further referral to physiotherapy according to the STarT back screening Tool classification, advice of promotion of activity, return to work and information of exercise venues and self-help groups, and educational video. Medium-risk patients were given treatment aiming to decrease symptoms and increase function. High-risk patients were given psychologically informed physiotherapy, ie, a cognitive–behavioral approach integrated with traditional physiotherapy with attention on both biomedical and psychosocial aspects of pain and function.	Assessment, advice, exercises and if needed a referral to further physiotherapy without any limitations of the content.	The intervention group had significantly lower disability compared to the control condition at 4- and 12-mo follow-ups.
Holm et al.,^28^ Sweden	Study the effects of a tailored behavioral medicine treatment compared with supervised physical exercises.	Patients with musculoskeletal pain >3 mo, aged 12–16 y, n = 32,	Functional behavior analysis on problematic behaviors in activities was formulated. Treatment was given in 3 tracks: (1) Strength, endurance, circulation, posture, range of motion, stabilization, coordination, aerobic exercises, and progressive relaxation; (2) Information and behavior change techniques for sleep, eating, and stress; (3) Standardized behavior change techniques (such as goal setting, feedback, self-monitoring, problem solving, distraction) to facilitate change in activities, self-efficacy, catastrophizing, anxiety, and fear of movement.	Strength, endurance, circulation, posture, range of motion, stabilization, coordination, aerobic exercises. Information about sleep, eating, and stress.	No significant between-group differences after Bonferroni correction. Both groups showed positive changes over time (posttreatment) in disability and pain intensity. The experimental group had larger effect sizes compared to control group over time. Seventy-five percent of the experimental group and 62% of the control group perceived themselves fully recovered.
Lotzke et al.,^34^ Sweden	Study the effects of a person-centered physical therapy rehabilitation program based on a cognitive–behavioral approach compared to conventional care.	Patients with degenerative low back disk disease before and after fusion surgery, 18–70 y of age, n = 118	Identify ability to stay active despite pain, increase knowledge regarding association with pain and activity-related behaviors, challenge cognitions and emotions in performing physical activity during a behavioral experiment, enhance the self-efficacy and form functioning- related goals, identify fear-avoidance beliefs, and revise goals for functioning in a booster session	In a single physiotherapy session, information about the postoperative mobilization and exercise program after surgery was given. Encouragement to stay active and start performing the recommended exercises before surgery was included.	No significant between group over time (6-mo follow-up) difference was shown in disability. A significant interaction effect was shown for the EQ-5D index in favor of the person-centered physiotherapy rehabilitation program based on a cognitive–behavioral approach.
Ludvigsson et al.^35^ and follow-up by Overmeer et al.,^43^ Sweden	Study the effect of neck-specific exercise (NSE) or neck-specific exercise with a behavioral approach (NSEB) compared to prescription of physical activity (PPA).	Patients with chronic whiplash-associated disorders in the age group of 18–63 y, n = 216	NSE: Neck-specific exercises, information of neck functioning, postural control, isometric and other progressive neck-specific exercises, home exercise, instructions to continue exercises. NSEB: As NSE + information of awareness of thoughts on behavior, activity-based goals for neck-specific exercises, breathing exercises, pacing, reinforcement of pain management education, and strategies for relapse prevention.	PPA: Physical examination, motivational interview, and prescription of individualized physical activity.	NSE and NSEB groups differed significantly in disability compared to PPA group at 3- and 6-mo follow-ups. No differences between NSE and NSEB groups. At 2-y follow-up, the NSEB group had maintained the over-time gains in disability in comparison to the NSE and PPA groups. Catastrophizing decreased significantly more in NSE (up to 1 y) and NSEB (up to 2 y) than in PPA. Kinesiophobia and anxiety decreased significantly more in NSE (up to 1 respectively 2 years) compared to NSEB and PPA.
O'Keeffe et al.,^40^ Ireland	Study the effects of an individualized cognitive functional therapy compared to a group-based exercise and education for individuals with chronic low back pain	Patients with chronic low back pain aged between 18 and 75 y, n = 206	Cognitive functional therapy including identification of multidimensional factors of relevance contributing to pain and disability, making sense of pain, exposure with control and supporting lifestyle change	Group-based pain education, relaxation, and exercise	Cognitive functional therapy significantly decreased disability, but not pain, at 6 and 12 mo compared with the control intervention.
Sandborgh et al.,^45^ Sweden	To investigate effects of tailored treatment targeted to 4 subgroups of patients with persistent musculoskeletal pain: low-risk patients with tailored intervention (experimental); low-risk patients with nontailored physical exercise (control); high-risk patients with tailored intervention (experimental); high-risk patients with nontailored acute or subacute (control).	Patients in primary health care with musculoskeletal pain for 4 wk, aged 18–65 y, n = 45	Tailoring of treatment to biopsychosocial and behavioral factors for both high- and low risk patients. Behavior goal identification, self-monitoring of behavior in activities, functional behavior analysis to identify behavioral skills necessary for goal achievement, apply the skills in complex behaviors, ie, cognitive and motor behaviors, and problem-solving strategies, skill generalization to daily activities	Individually adapted and structured physical exercise.	Tailored treatment was partially superior to physical exercise treatment. No posttreatment differences in pain-related disability but for higher-rated global outcomes for tailored group (performance of daily activities and confidence in handling future risk situations). Targeting by treatment dosage was effective for low-risk patients.
Sterling et al.,^51^ Australia	Study effects of a stress inoculation training integrated with exercise compared to exercise only.	Patients with acute whiplash-associated disorders, aged 18–65 y, n = 108	Educational self-management guide, exercises according to guidelines for acute whiplash-associated disorders, return to normal activities, manual therapy was allowed. Teach strategies to identify and manage acute stress responses.	Educational self-management guide, exercises according to guidelines for acute whiplash-associated disorders, return to normal activities, manual therapy was allowed.	Stress inoculation training with exercise decreased disability significantly more than exercise only at 6-wk, and 6- and 12-mo follow-ups.
Söderlund et al.,^49^ Sweden	Study the effects of physiotherapy management complemented with cognitive–behavioral components compared with standard physiotherapy for patients with chronic WAD	Patients with chronic whiplash-associated disorders, aged between 18 and 65 y, n = 33	Functional behavioral analyses of problem behaviors in daily activities. Goal setting for changing behaviors. Learning basic physical and psychological skills, applying basic skills in daily activities, and strategies for maintenance of the skills.	Stabilization, stretching, coordination of neck and shoulder muscles, body posture and arm muscle strength exercises at home and gym. Pain relief treatments: Relaxation, transcutaneous electric stimulation, and acupuncture could be included	No differences between the groups over time in disability, pain intensity, or in physical measures. At 3 mo follow-up, the experimental group's ability in daily activities was significantly better compared to control group. The experimental group showed better long-term compliance, ie, they used the learned skills to manage or prevent neck pain in daily life significantly more often than control group.
Vibe Fersum et al.,^56,57^ Norway	Study the effects of a classification-based cognitive functional therapy (CFT) compared with manual therapy and exercise (MT-EX) for the management of nonspecific chronic low back pain.	Patients with chronic low back pain, aged 18–65 y, n = 121	Cognitive functional therapy including identification of multidimensional factors of relevance contributing to pain and disability, making sense of pain, and exposure with control.	Joint mobilization or manipulation techniques, motor control exercise program	At 1-y follow-up, the CFT group showed lower disability, pain intensity, anxiety and depression, and fear-avoidance levels compared to MT-EX group. The results were maintained at the 3 y follow-up except in pain intensity.
Wiangkham et al.,^58^ United Kingdom	Study the feasibility and patient outcomes of active behavioral physiotherapy intervention (ABPI) compared to standard physiotherapy intervention	Patients with chronic whiplash-associated disorders, aged 22–70 y, n = 28	ABPI has 4 phases facilitating understanding (information, simple tasks, challenge, evaluation, feedback), maturity (improve information, variation of tasks, challenge, evaluation, feedback), stamina (maintain motivation, complex tasks, challenge, evaluation, feedback), and coping (increase self-efficacy in self-management, encourage to healthy lifestyle, evaluation, feedback). Techniques such as exercise, relaxation, and manual therapy could be included.	Reassurance, education, manual therapy, exercise therapy and physical agents, as well as a home exercise program	Descriptive statistics three mo after baseline support the positive effect of ABPI compared to standard physiotherapy in: disability, quality of life, neck range of motion, and pressure pain threshold; but not in 2 psychological outcomes.
Åsenlöf et al.^4^ and follow-up by Emilson et al.,^19^ Sweden	Study the effect of an individually tailored behavioral medicine intervention in physiotherapy compared to physical exercise therapy.	Patients with chronic musculoskeletal pain (>4 wk), aged 18–65 y, n = 122	Tailored behavioral medicine approach in physiotherapy. The intervention consisted of 7 phases: (1) Behavior goal identification; (2) self-monitoring of behavior in activities; (3) functional behavior analysis to identify the physical, cognitive, and behavioral skills necessary for goal achievement; (4) physical and cognitive basic skills acquisition; (5) apply the skills in complex behaviors, ie, cognitive and motor behaviors, and problem-solving strategies; (6) skill generalization to daily activities; (7) maintenance and relapse prevention, and problem-solving strategies.	Physical exercises according to problems related to physical impairment goal; joint mobility, strength, endurance, balance, and coordination.	Significant difference between groups over time in disability, pain intensity, pain control, fear of movement, and higher performance in daily activity performance up to postintervention but not up to 3-mo follow-up. At 10-y follow-up, a significant difference was shown in sick leave in favor to the individually tailored behavioral medicine intervention.

Table 4 for the implementation in clinical practice trials includes the following: reference, country, aim, target group for the implementation and context, patient sample for the intervention, intervention implemented on the patients, control intervention implemented on the patients, and the patient outcomes.

**Table 4:** Characteristics of included studies of implementation of a behavioral medicine approach in physiotherapists' clinical practice for patients with musculoskeletal pain.

Reference, country	Aim	Target group for implementation and context	Patient sample for intervention	Implemented intervention on patients	Implemented control intervention on patients	Results of patient outcomes
Fritz et al.,^23^ Sweden	To explore how an intervention to facilitate the implementation of a behavioural medicine approach in primary health care improves the health outcomes of patients with persistent musculoskeletal pain.	Physiotherapists in primary health care, n = 24.	Patients with chronic musculoskeletal pain (>4 wk), aged 18–65 y, n = 155.	Identifying and managing cognitive, emotional, social, physical, and lifestyle barriers of importance for the target behaviour change. Behaviour change techniques: patient's goal-setting, self-monitoring of behaviour, the setting of graded tasks, problem solving, feedback on the patient's behaviours, and maintenance strategies.	Standard care.	No differences between the experimental and control groups over time (pre, post, 6, 12 m) regarding pain-related disability, pain intensity, and self-rated health. Significant improvements over time in both the experimental and control groups and the effect sizes were medium to large. The percentage of patients on sick leave decreased significantly in the experimental group but not in the control group.
Overmeer et al.,^42^ Sweden	The aim of this study was to investigate the effects on patients' outcomes of an 8-d university-based training course, aimed at identifying and addressing psychosocial prognostic factors during physiotherapy treatment for patients with musculoskeletal pain compared to physiotherapist on a waiting list for the same course.	Physiotherapists in an outpatient and inpatient setting, n = 42	Patients with acute or subacute musculoskeletal pain, aged 18–65 y, n = 229	Treatment according to content of the course: Identify “yellow flags”; behavioral medicine principles and cognitive–behavioral management strategies; physical examination and information to the patient from a biospsychosocial perspective; reassurance; and identify and manage fear avoidance	Standard care.	No significant differences in pain or disability between the groups were found.
Reid et al.,^44^ USA	Study the effectiveness of a cognitive–behavioral pain self-management (CBPSM) compared with usual care (UC) for older adults receiving home care.	17 home care rehabilitation teams each including at least 15 PTs (totally 255 PTs).	Patients with activity limiting pain, aged >55 y, n = 588	Cognitive behavioral pain self-management: Pain, activity and sleep education, goal setting, relaxation, imagery (as a relaxation technique), pleasant activity scheduling and activity pacing, managing flare-ups (specific techniques), and problem solving regarding sleep. Booklet to reinforce the CBSM, reminders to practice learned techniques between physiotherapy sessions.	Usual care: evaluation of physical and psychological functioning, home environment, need/use of assistive devices, therapy goals by a physician, individualized exercise programs aiming to improve strength, range of motion, balance, coordination, gait, activities of daily life functioning, and reduce fall risk.	CBPSM and UC significantly decreased in disability and all other reported outcomes (pain intensity, ADL limitations, gait speed, depressive symptoms, and pain self-efficacy). No between group over time differences were found in any of the outcomes at 2-mo follow-up.

Table 5 shows that (1) randomized controlled effect trials and (2) implementation in clinical practice trials both contributed to the content and included the following variables: integrated psychosocial aspects, integrated behavioral aspects, integrated biomedical/physical aspects, behavior change techniques (explicitly reported in the study or interpreted by the authors of the present scoping review), and cluster categorizing of behavior change techniques.

**Table 5:** The studies' experimental interventions' matching with the definition of behavior medicine in physiotherapy: “Integration of psychosocial, behavioral, and biomedical knowledge in analyses of patients'/clients' behaviors in activities of importance for participation and in choosing and applying treatment and behavior change methods and evaluating outcomes”^47^ are presented.

Randomized controlled effect trials	Integrated psychosocial components	Integrated behavioral components	Integrated biomedical/physical components	Behavior change techniques, explicitly reported in the study or interpreted by the authors of the present scoping review	Cluster categorizing of behavior change techniques
Archer et al.^3^	Cognitive restructuring	Behavioral self-management	Advice of lifting and driving, relaxation, activity level	Advice, education, graded activity plan, problem solving, goal setting, distraction, replace negative thoughts, balancing rest and activities, and maintenance strategies	Repetition and substitution Antecedents Natural consequences Feedback and monitoring Goals and planning Shaping knowledge Other
Bring et al.^6^	Functional behavioral analysis and everyday activity goals regarding psychological and social skills. Treatment included basic skills in psychological and social area, and psychological and social skills applied in daily activities.	Functional behavioral analysis for specifying behavioral skills and goals, all skill rehearsal in daily activities.	Functional behavioral analysis (regarding physical skills). Treatment included basic skills in physical area, physical skills applied in activities	Self-monitoring of behavior, behavior goal setting, rehearsal of physical, cognitive and behavioral skills, rehearsal of self-management skills, strategies for maintenance, and relapse prevention.	Repetition and substitution Goals and planning Feedback and monitoring Shaping knowledge Other
Cederbom et al.^8^	Individual functional behavior analysis of psychological, social, and physical environmental factors.	Functional behavior analysis for specifying skills needed.	Individual functional behavior analysis of physical factors. Treatment included advice on physical activity and its benefits and exercise program.	Advice, goal setting, self-monitoring, feedback, problem-solving strategies, strategies for maintenance and relapse prevention.	Natural consequences Feedback and monitoring Goals and planning Shaping knowledge Other
Cederbom et al.^9,10^	Analyses of the psychological, social, and environmental factors related to the goal behaviors and treatment goals. Improve cognitive, or social skills, improve self-efficacy, decrease fear of falling and fear of movement in the goal behavior.	Improve behavioral skills, and generalize skills to other behaviors.	Analyses of the physical factors related to the goal behaviors and treatment goals. Improve physical skills. Advice on and increase of physical activity and its benefits, and functional exercises.	Self-monitoring of goal behavior and physical activity, goal setting, problem-solving strategies, and strategies to maintain new behavior.	Natural consequences Feedback and monitoring Goals and planning Shaping knowledge Other
Hill et al.^27^ and treatment description by Main et al.^36^	Identifying relationships between beliefs, expectations, distress, and pain behaviors.	Facilitate self-disclosure, patient-centered approach for building confidence and increasing self-efficacy, problem solving with pain management techniques	Exercise, and increase in function	Information, education, challenge unhelpful beliefs, goal setting, pacing, graded activity, reshape expectations, problem solving, and relapse prevention	Repetition and substitution Natural consequences Feedback and monitoring Goals and planning Shaping knowledge Other
Holm et al.^28^	Identification of levels on and improving self-efficacy, catastrophizing, anxiety, and fear of movement.	Functional behavior analysis on problematic behaviors in activities. Improve behavioral skills.	Strength, endurance, circulation, posture, range of motion, stabilization, coordination, aerobic exercises, and progressive relaxation.	Goal setting, feedback, self-monitoring, problem solving, distraction, and prompts. Information and behavior change techniques for improving sleep, eating, and stress.	Antecedents Associations Natural consequences Feedback and monitoring Goals and planning Shaping knowledge
Lotzke et al.^34^	Enhance self-efficacy, and identify fear-avoidance beliefs.	Challenge cognitions and emotions in performing physical activity during behavioral experiments.	Identify ability to stay active despite pain	Increase knowledge of pain and behaviors, form functioning-related goals, and revise goals in a booster session	Associations Natural consequences Goals and planning Shaping knowledge
Ludvigsson et al.^35^ and follow-up by Overmeer et al.^43^	Information of awareness of thoughts and beliefs in behavior.	Alternate between activities and rest (pacing).	Neck-specific exercises, information of neck functioning, postural control, isometric and other progressive neck-specific exercises, home exercise, instructions to continue exercises, and breathing exercises.	Awareness of thoughts and beliefs in behavior, activity-based goal setting, pacing, reinforcement of pain management education, and strategies for relapse prevention.	Natural consequences Feedback and monitoring Goals and planning Shaping knowledge Other
O'Keeffe et al.^40^	Identification of pain provocative and modifiable cognitive factors, coping strategies, fear of pain beliefs, and making sense of pain. Cognitive component in treatment focus on information and discussion of associations between pain and beliefs.	Identification of the lifestyle behaviors, avoidance of activities, stress response, exposure with control, and supporting lifestyle change. Functional integration into avoided daily activities. Social reengagement.	Identification of pain provocative and modifiable movement-related factors and functional impairments. Normalize postural and movement behaviors, and enhance body awareness and control. Physical activities gradually increased.	Information, motivational interviewing techniques, goal setting, graded activity, exposure, and mindfulness skills.	Repetition and substitution Antecedents Associations Natural consequences Feedback and monitoring Goals and planning Shaping knowledge
Sandborgh et al.^45^	Identification of negative pain beliefs and cognitions Targeting for low-or high risk for disability. Tailoring treatment to biopsychosocial factors.	Assessment of behaviours in everyday physical activities. Targeting and tailoring treatment behavioural factors. Basic and applied behaviour skill training in everyday activities	Assessment of physical factors of importance for behavioural goals Tailored physical exercise	Behavioral goal setting, self-monitoring, evaluation of performance, training of basic biopsychosocial skills. Recognition of negative thoughts and positive self-statements, reinforcing feedback. Merging basic skills into complex behaviours, problem-solving skills, generalization of skills, and strategies for maintenance and relapse prevention.	Repetition and substitution Associations Natural consequences Feedback and monitoring Goals and planning Social support Self-belief Shaping knowledge Other
Sterling et al.^51^	Identify emotional and cognitive stressors	Identify stress and stressors affecting pain, behavior, and emotions, physical performance, and cognitions. Stress management skills applied in different situations, develop confidence, and tolerance	Exercise, return to activities, and relaxation	Information, graded exercise, problem solving, coping strategies, and generalization of skills	Natural consequences Repetition and substitution Goals and planning Self-belief
Söderlund et al.^49^	Identification of negative pain beliefs and cognitions. Psychological basic and applied skills training: Change of negative pain beliefs and cognitions, coping with pain and increasing functional self-efficacy in daily activities.	Functional behavioral analyses of problem behaviors in daily activities. Physical and psychological skill rehearsal was integrated within daily activities.	Physical basic skill training: Relaxation, reeducation of cervicothoracic posture, increase of neck range of motion, coordination, and endurance of neck and shoulder muscles.	Self-monitoring of behavior, skills rehearsal, goal setting, graded activity, skills generalization in daily activities, feedback, distraction for negative pain beliefs and cognitions, problem-solving strategies, and reevaluation of goals. Plan for risk situations for relapse and maintenance of the new behaviors in daily activities.	Repetition and substitution Antecedents Associations Natural consequences Feedback and monitoring Goals and planning Self-belief Shaping knowledge Other
Vibe Fersum et al.^56,57^	Identification of pain provocative and modifiable cognitive factors, coping strategies, fear of pain beliefs, and making sense of pain. Cognitive component in treatment focus on information and discussion of associations between pain and beliefs.	Identification of the lifestyle behaviors, avoidance in activities, and exposure with control. Functional integration into avoided daily activities. Self-management practices.	Identification of pain provocative and modifiable movement-related factors and functional impairments. Normalize postural and movement behaviors, and enhance body control. Physical activities gradually increased.	Goal setting, graded activity, exposure, and relapse-prevention plan.	Associations Natural consequences Goals and planning Shaping knowledge Other
Wiangkham et al.^58^	Increase self-efficacy in self-management. Facilitate motivation and relaxation.	Facilitate healthy lifestyle behavior. Stress management.	Return to normal function/movement, specific exercise programs for stability and mobility, postural control, and advice to act as usual. Whiplash education.	Increase self-efficacy in exercise by verbal persuasion, education, promote stress self-management, feedback, reassurance, and progressive exercises. Facilitate the adoption/maintenance of a healthy lifestyle.	Repetition and substitution Natural consequences Feedback and monitoring Shaping knowledge Other
Åsenlöf et al.^4^ and follow-up by Emilson et al.^19^	Functional behavior analysis to identify cognitive skills necessary for goal achievement, and cognitive basic skill acquisition.	Behavior goal identification, self-monitoring of behavior in activities, functional behavior analysis to identify behavioral skills necessary for goal achievement, apply the skills in complex behaviors, ie, cognitive and motor behaviors, and problem-solving strategies, and skill generalization to daily activities	Functional behavior analysis to identify the physical skills necessary for goal achievement, and physical and basic skill acquisition.	Self-monitoring of behavior, goal setting, rehearsal of skills, feedback, reevaluation of goals, integration of skills in complex behaviors, generalization of skills, maintenance and relapse prevention, and problem-solving strategies.	Repetition and substitution Antecedents Associations Natural consequences Feedback and monitoring Goals and planning Self-belief Shaping knowledge Other
**Implementation in clinical practice trials (implemented intervention on patients)**	**Integrated psychosocial components**	**Integrated behavioral components**	I**ntegrated biomedical/physical components**	**Behavior change techniques, explicitly reported in the study or interpreted by the authors of the present scoping review**	**Cluster categorizing of behavior change techniques**
Fritz et al.^23^	Asking about psychological and environmental factors, discussing physical and social environmental change, and psychological exercises.	Asking about daily activities, and practicing daily activities.	Physical examination and exercises.	Patient's goal-setting, self-monitoring of behaviour, the setting of graded tasks, problem solving, feedback on the patient's behaviours, positive reinforcement, self-reinforcements, prompts, and maintenance strategies.	Associations feedback and monitoring Goals and planning Shaping knowledge Other
Overmeer et al.^42^	Treatment according to content of the course: Identify “yellow flags,” information to the patient from a biospsychosocial perspective.	Treatment according to content of the course: behavioral medicine principles and cognitive–behavioral management strategies	Treatment according to content of the course: physical examination and information to the patient from a biospsychosocial perspective.	Information, reassurance, and identify and manage fear avoidance	Repetition and substitution Associations Natural consequences Feedback and monitoring
Reid et al.^44^	Relaxation, imagery (as a relaxation technique)	Cognitive behavioral pain self-management.	Pain, activity and sleep education, relaxation, and activity pacing.	Pain, activity and sleep education, goal setting, pleasant activity scheduling and activity pacing, managing flare-ups, and problem solving. Reinforcer and reminder in booklet form.	Repetition and substitution Associations Natural consequences Goals and planning Feedback and monitoring

Reported behavior change techniques and categorizing of the techniques according to Michie et al.^38^ are also presented, separately for the (1) randomized controlled effect trials and (2) implementation in clinical practice trials. The content of the cluster categories for the behavior change techniques according to Michie et al.^38^ The contents are presented based on in the included studies explicitly reported, or interpreted by the authors of the present scoping review, behavior change techniques: Repetition and substitution: Behavior substitution, graded tasks, behavioral rehearsal, generalization of target behavior; antecedents: restructuring physical environment, distraction; Associations: Exposure, classic conditioning, prompts/cues; Natural consequences:Information of natural health consequences; Feedback and monitoring: Feedback on behavior, self-monitoring of outcome of behavior, self-monitoring of behavior; Goals and planning: Action planning, problem solving/coping planning, goal setting outcome and behavior, review of goals; Social support: Social support practical; Self-belief: Focus on past success, verbal persuasion to boost self-efficacy; Shaping knowledge: Antecedents, behavioral experiments, instruction of how to perform behavior; Other: Strategies for managing relapse and maintenance of behavior.

### 2.5. Synthesis of results

A critical quality appraisal was not conducted. The studies were grouped according to the 2-fold aim of this study: (1) randomized controlled effect trials and (2) implementation in clinical practice trials. All included study characteristics, listed in detail above in data charting process and parameters section, are summarized in Tables 3 and 4. The matching of the study interventions with the definition of behavior medicine in physiotherapy^47^ was performed from the integration of psychosocial, behavioral, and biomedical knowledge perspective for (1) randomized controlled effect trials and (2) implementation in clinical practice trials separately and is presented in Table 5. This synthesis required an analytical interpretation of the intervention contents to determine whether the content fit with the psychosocial, behavioral, and biomedical knowledge integration. In the studies clearly reported or by the current scoping review authors interpreted behavior change techniques were categorized according to the taxonomy by Michie et al.^38^ and are presented in Table 5.

## 3. Results

The results are presented both separately and together for the (1) randomized controlled effect trials and (2) implementation in clinical practice trials.

### 3.1. Selection of sources of evidence

**Figure 1.**
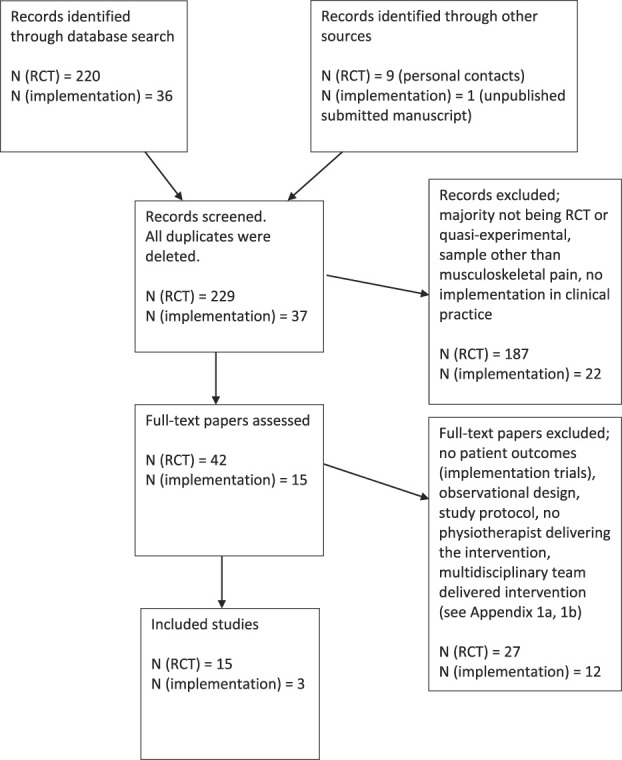
PRISMA chart for the study selection. N (RCT) refers to the randomized controlled effect trials. N (implementation) refers to the implementation in clinical practice trials.

Five databases were searched; see Table 1 (randomized controlled effect trials) and Table 2 (implementation in clinical practice trials) for the search strategy, the terms, and number of eligible studies from each database. Figure 1 presents the PRISMA chart for the study selection, number of studies in each phase of the selection process, and major reasons for study exclusion; see also appendix for excluded full-text articles (available online as supplemental digital content at http://links.lww.com/PR9/A75). In total, 18 studies, 15 randomized controlled effect trials and 3 trials of implementation in clinical practice, were included in this scoping review.

### 3.2. Characteristics and results of individual sources of evidence

Studies that contributed to the results were from Australia,^51^ Ireland,^40^ Norway,^9,10,56,57^ Sweden,^4,6,8,19,23,28,34,35,42,43,45,49^ the United Kingdom,^27,58^ and the United States.^3,44^

Target groups for the patient interventions varied. One study was targeted at adolescents,^28^ 3 were targeted at older persons,^8–10,44^ and the rest of the studies were targeted at people of working age.^3,4,6,19,23,27,34,35,40,42,43,45,49,51,56–58^

The patient outcomes showed mostly significant differences in favor of the experimental intervention in the randomized controlled effect trials. The results for the short-term effects (3-month follow-up) were mixed, but the long-term effects were better than those of the control condition. Six studies had 12 months of follow-up,^6,27,35,40,51,56^ one had 24 months,^43^ one had 36 months,^57^ and one had 10 years.^19^ The study characteristics, experimental and control interventions, and patient outcomes of the included randomized controlled effect trials regarding investigations of a behavioral medicine approach in physiotherapy for patients with musculoskeletal pain are presented in Table 3.

The 3 implementation in clinical practice trials showed no significant differences in short-term follow-up. One of these studies^23^ reported a 12-month follow-up showing a difference in the percentage of patients on sick leave in favor of the experimental group. The study characteristics, implemented interventions, and patient outcomes of the included studies that implemented a behavioral medicine approach in a physiotherapists' clinical practice among patients with musculoskeletal pain are presented in Table 4.

### 3.3. Synthesis of the results for the matching of the interventions with the definition of behavioral medicine in physiotherapy

Both the randomized controlled effect trials and the implementation in clinical practice trials mostly integrated psychosocial, behavioral, and biomedical/physical aspects in the experimental patients' intervention condition, except in one study. The study by Ludvigsson et al.^35^ did not clearly report the integration of behavioral aspects in the experimental intervention except that pacing as a behavior change technique was included, implying that patients were learning to alternate between activities and rest. The majority of behavior change techniques reported in randomized controlled effect trials were categorized^38^ as “information of natural consequences,” “feedback and monitoring,” “goals and planning,” and “shaping knowledge,” and the majority of techniques reported in the implementation in clinical practice trials were categorized^38^ as “information of natural consequences,” “feedback and monitoring,” and “goals and planning.”

All included randomized controlled effect trials and the implementation in clinical practice trials matched the definition of behavioral medicine in physiotherapy.

The results for matching the experimental interventions of the studies with the definition of behavior medicine in physiotherapy, ie, the “integration of psychosocial, behavioral and biomedical knowledge in analyses of patients'/clients' behaviors in activities of importance for participation and in choosing and applying treatment and behavior change methods and evaluating outcomes”^47^ are presented in Table 5. Table 5 also includes studies reporting behavior change techniques and the categorization of the techniques according to Michie et al.^38^

## 4. Discussion

### 4.1. Summary of evidence

Synthesis of the results for the matching of the patient interventions with an existing definition of behavioral medicine in physiotherapy for the randomized controlled effect and the implementation in clinical practice trials showed that the interventions mostly integrated psychosocial, behavioral, and biomedical/physical aspects, and thus, showed conformity with the existing definition of behavioral medicine in physiotherapy.^47^ The reported behavior change techniques in all trials were few and commonly occurred in the categories^38^ “"information of natural consequences,” “feedback and monitoring,” and “goals and planning.” The patient outcomes in the randomized controlled effect trials for the long-term follow-ups showed mostly positive effects in comparison to the control condition. The implementation in clinical practice trials reported no differences in the short term.

The matching of intervention components with the definition of behavioral medicine in physiotherapy was somewhat difficult due to overlap between the psychosocial and behavioral knowledge or components/aspects to be integrated in the assessment, treatment, and evaluation of patients with musculoskeletal pain. Because a behavior can be defined as movement or activities or as the cognitive, emotional, or physiological response of an individual,^52^ it is easy to see how behavioral aspects can spill over to the psychosocial and perhaps physical aspects of the definition of behavioral medicine and can thus be difficult to categorize. The definition of behavioral medicine in physiotherapy actually demands the integration of the behavioral, psychosocial, and physical aspects during both analysis and treatment, which was difficult to identify in the included studies. Frequently, the integration was described thoroughly for the analysis but not clearly for the treatment, ie, how the physiotherapists took into account the results of the analysis in the treatment. For example, the identification of fear of movement was mentioned but how to manage this fear in the treatment was not.^9,10,34^ Similar results were shown in a recent systematic review in which it was not possible to identify how the cognitive–behavioral components used in physiotherapy were actually operationalized.^26^ The reported intervention components in this study varied quite a lot. Frequently reported components (Table 5) were improve self-efficacy and reduce fear and catastrophizing, generally discuss pain beliefs, increase activities and pain self-management strategies, improve stress management, rehearsal of behaviors in daily activities, facilitate exercise, and improve function, but several other components had also been used. Interventions and their components in the included studies have certainly been thoughtfully developed. However, it might be possible to further refine the interventions by, eg, using the behavior change wheel system and its underpinning framework for systematically designing interventions.^37^ The behavior change wheel relies on 3 main functions: capability (optimize psychological and physical abilities and thus enabling the behavior), opportunity (optimize social and physical environment to enable the behavior), and motivation (balancing activating and inhibiting mechanisms of the behavior).^5,37^ The systematic use of, eg, the behavior change wheel in future studies could also lead us to more uniform definition of behavioral medicine interventions in physiotherapy framework. Thus, questions for research remain regarding for which patients, which components, in which order, and how many components need to be integrated to maximize patient outcomes. In addition, adherence to the intervention is important, ie, facilitating treatment integrity must be considered when designing interventions and their components for implementing in physiotherapy with a behavioral medicine approach. Being able to integrate the behavioral, psychosocial and physical aspects in assessment, analysis, and treatment and to adhere to a complex treatment approach, such as that in behavioral medicine, in both research context and clinical practice demands versatile and often new practical skills and knowledge^22,24,41^ as well as new competencies in clinical reasoning.^15,17^ In addition, barriers, eg, time, staff, and monetary resources,^21^ as well as support from managers^1,53^ in carrying out implementation in clinical practice trials and following patients in the long term must be considered in intervention designs.

The most common behavior change techniques in the included trials were in the categories “information of natural consequences,” “feedback and monitoring,” and “goals and planning.”^38^ “Information of natural consequences” included information on health consequences; “feedback and monitoring” included giving feedback on behavior, self-monitoring of behavior, and the outcome of behavior; and “goals and planning” included action planning, problem solving/coping planning, goal setting for outcome and behavior, and reviewing goals. The major reported categories include many more behavior change techniques^38^ that were not used in the studies. This suggests that the patient outcomes in these studies might have been positively different if more techniques were used, as previously concluded.^20,32^ The low number of behavior change techniques used in the included studies might be due to limited knowledge about the techniques that could have been chosen.^31^ Beyond the major categories, 7 others were identified; however, they were presented only in a few studies.^3,4,40,46,49^ Why the behavior change techniques were chosen for the respective studies was not clearly stated in any of the studies. This can be associated with the fact that the results of the integration of the behavioral, psychosocial, and physical aspects in the analysis of behavior during activities could not clearly be identified in the treatment, ie, the translation of the results from the behavior analyses to the treatments was not obvious. Reasons for the purposeful selection of behavior change strategies for the treatment should be focused on future studies.

Nine randomized controlled effect trials^6,19,27,35,40,43,51,56,57^ had follow-ups between 1 and 10 years and were able to show more stable positive experimental intervention effects across the studies. Interventions aiming to change the behavior of the patient most likely need to have long-term follow-ups to show the actual effects of the techniques used to change the pain behaviors. Regarding the 3 implementation in clinical practice studies, only one study^23^ reported 1-year follow-up with the difference shown in the decreased percentage of patients requiring sick leave in the behavior medicine intervention. However, this study has not yet been published and thus demands caution when drawing conclusions.

### 4.2. Limitations and strengths

A scoping review has both limitations and strengths. A scoping review can be seen as a preliminary assessment of available studies on the topic in question. One of the strengths is that ongoing research can be included in the review. However, this kind of research should be at least submitted for peer-review to ensure that the data collection and results have been finalized. Including research in progress can also be a limitation because conclusions from that kind of articles are not final as they are for articles that have been accepted for publication and completed a peer-review process. A limitation can also be that we did not register this scoping review, which implies that eg, the transparency of our research was not optimal. Thus, other researchers' input regarding, eg, search strategy was not possible and could have influenced the results. Further limitations of the current scoping review are the nonexistent quality assessment^11,33^ and conclusions that are only descriptively summarized, which can have negative impact on the reliability of the results. However, the main aim of this review was not to investigate the effects of any intervention, where the quality assessment is very important. The main aim was to describe how the interventions matched with a definition of behavioral medicine in physiotherapy and to categorize the reported behavior change techniques for patients with musculoskeletal pain with the goal of informing future research, policies, and practice.

The search terms related to the behavioral medicine approach in physiotherapy may have biased the results because there was a large variation in the terms that could be chosen. The screening of the search results for inclusion was done by one person, which may have biased the results. However, in case of uncertainties, the study was included to the next step where full-text articles were assessed by all authors leading to agreement of final inclusion. Another limitation is that only search terms in English were used, therefore potentially relevant studies in other languages may have been excluded. In addition, the analysis of the matching of the interventions with the behavioral medicine definition may not be complete because there might exist several intervention components and behavior change techniques that were not explicitly reported by the authors of the included studies. However, the current article is a scoping review and thus does not try to give an appearance of being a comprehensive systematic review with associated meta-analysis or meta-synthesis. One more strength of this study was the use of a previously published definition of the behavioral medicine in physiotherapy, with which the included studies were matched, thus giving us quite a clear platform for comparing the interventions and definition.

## 5. Conclusion

The synthesis of the results for the matching of the patient interventions with an existing definition of behavioral medicine in physiotherapy for the randomized controlled effect and the implementation in clinical practice trials showed that the interventions mostly integrated psychosocial, behavioral, and biomedical/physical aspects, and thus complied with the existing definition of behavioral medicine in physiotherapy. The reported behavior change techniques were few and were commonly in the categories “information of natural consequences,” “feedback and monitoring,” and “goals and planning.” The short-term patient outcomes were mixed, but the long-term follow-ups showed mostly positive effects.

## 6. Future challenges

The goal of this scoping review was to possibly inform future research, policies, and practice and not to explicitly, critically review effects for any evidence. The future challenges are, eg, that we need to keep refining interventions integrating psychosocial, behavioral, and biomedical/physical aspects, carefully choose the components and techniques that should be included and further develop these for physiotherapeutic purposes, but also, how to implement the components and techniques efficiently when integrating behavioral medicine in physiotherapy. Furthermore, beyond challenges for integrating psychosocial, behavioral, and biomedical/physical aspects, support on organizational and leadership levels in the implementation of behavioral medicine in physiotherapy should also be investigated to find the optimal contextual aspects for effective implementation.

## Disclosures

The authors have no conflicts of interest to declare.

## Appendix A. Supplemental digital content

Supplemental digital content associated with this article can be found online at http://links.lww.com/PR9/A75.
